# CONSTRUCT VALIDITY OF EXERCISE ADHERENCE RATING SCALE IN COMMUNITY DWELLING OLDER WOMEN: PROOF OF CONCEPT

**DOI:** 10.1093/geroni/igac059.2893

**Published:** 2022-12-20

**Authors:** Helen Graham, Judith Scott, Melissa Benton

**Affiliations:** University of Colorado Colorado Springs, Colorado Springs, Colorado, United States; University of Colorado Colorado Springs, Colorado Springs, Colorado, United States; University of Colorado Colorado Springs, Colorado Springs, Colorado, United States

## Abstract

Exercise is an effective health promotion strategy that has a positive effects on frailty and sarcopenia, particularly in older women, yet they do not adhere to exercise guidelines. Accurate assessment is critical for clinical management and research with this population. Interviews and exercise logs are most commonly used to assess adherence to exercise. However, they are burdensome and not highly reliable. A brief validated measure of exercise adherence for assessment is greatly needed. The six-item Exercise Adherence Rating Scale (EARS) was originally validated among a clinical orthopedic population but not been used in community-dwelling adults. Our aim was to determine the feasibility of using the EARS to assess exercise adherence among community-dwelling older women. Twelve women (age=72±7 years) completed one-time assessment for this proof-of-concept study. For the EARS, overall Cronbach’s alpha was 0.78 with inter-item correlations of 0.72–0.89 for all but item three that pertains to healthcare professionals (correlation of 0.27). When item three was removed, overall Cronbach’s alpha increased to 0.80. Correlation analysis with the reduced five-item scale demonstrated good construct validity. There were significant relationships with global quality of life (r=0.76, p < .01) and physical function (r=0.61, p < .05). There were also moderate to strong relationships with variables expected to be linked to exercise adherence including mobility (r=0.57), BMI (r=0.51), vigorous (r=0.40) and moderate (r=0.36) exercise, upper body strength (r=0.41) and lower body strength (r=0.30). Based on these findings use of a five-item version of the EARS is feasible and appears to be a valid measure of exercise adherence.

